# A Novel Prediction Model for Significant Liver Fibrosis in Patients with Chronic Hepatitis B

**DOI:** 10.1155/2020/6839137

**Published:** 2020-07-08

**Authors:** Yaqiong Chen, Jiao Gong, Wenying Zhou, Yusheng Jie, Zhaoxia Li, Yutian Chong, Bo Hu

**Affiliations:** ^1^Department of Laboratory Medicine, Third Affiliated Hospital of Sun Yat-sen University, Guangzhou, China; ^2^Department of Infectious Diseases, Key Laboratory of Liver Disease of Guangdong Province, Third Affiliated Hospital of Sun Yat-sen University, Guangzhou, China

## Abstract

**Background:**

Preventing liver fibrosis from progressing to cirrhosis and even liver cancer is a key step in the treatment of chronic hepatitis B (CHB). This study is aimed at constructing and validating a new nomogram for predicting significant liver fibrosis (*S* ≥ 2) in CHB patients.

**Methods:**

The nomogram was based on a retrospective study of 252 CHB patients. The predictive accuracy and discriminative ability of the nomogram were evaluated by the area under receiver operating characteristic curve (AUROC), decision curves, and calibration curve compared with the fibrosis 4 score (FIB-4) and aspartate aminotransferase-to-platelet ratio index (APRI). The results were validated using bootstrap resampling and an external set of 168 CHB patients.

**Results:**

A total of 420 CHB patients were enrolled based on liver biopsy results. Independent factors predicting significant liver fibrosis were laminin (LN), procollagen type III N-terminal peptide (PIIINP), and blood platelet count (PLT) in a multivariate analysis, and these factors were selected to construct the nomogram. The calibration curve for the probability of significant liver fibrosis showed optimal agreement between the prediction from the nomogram and actual observation. The prediction from the nomogram was more consistent with the results of liver biopsy than FIB-4 and APRI. The AUROC of the nomogram was higher than that of FIB-4 and APRI for predicting significant liver fibrosis. These results were confirmed in the validation set. Furthermore, the decision curve analysis suggested that the most net benefits were provided by the nomogram.

**Conclusions:**

We found the proposed nomogram resulted in a more accurate prediction of significant liver fibrosis in CHB patients and could provide the most net benefits. We recommend this noninvasive assessment for patients with liver fibrosis to avoid the risk of liver biopsy and earlier intervention to prevent the development of cirrhosis or liver cancer.

## 1. Introduction

In China, CHB is the primary cause of liver-related morbidity and mortality. Chronic hepatitis has been shown to lead to liver fibrosis, which may ultimately progress to liver cirrhosis, end-stage liver disease, and liver cancer [1]. Liver fibrosis, a dynamic pathological process, is an important sequel of chronic inflammatory liver disease. Different stages of liver fibrosis can influence clinical strategies. With an early diagnosis and the advent of effective antiviral therapies, the prognosis of CHB, even when presenting with histologically advanced fibrosis or cirrhosis, can be improved significantly, with a concomitant gain in the patients' quality of life [2]. Therefore, it is of great importance to find an inexpensive and more accurate scoring system for the early prediction and risk assessment of liver fibrosis stages in CHB patients, as it could potentially prevent the progression of HBV-related diseases.

Liver biopsy is the traditional gold standard for the assessment of liver fibrosis [3]. However, liver biopsy has some limitations, such as invasiveness, risk of serious complications, sampling error, and small sample size [4–6]. These limitations encourage us to investigate noninvasive and reliable approaches for the assessment of fibrosis.

Serum biomarkers, as attractive alternatives for assessing liver fibrosis, have many advantages of being inexpensive, readily accessible, noninvasive, and reproducible and can be obtained from almost all the patients in hospitals [7]. Currently, many serum biomarkers and panels have been studied for the assessment of fibrosis [8–10], such as hyaluronic acid (HA), PIIINP, LN, type IV collagen (IVC), FIB-4, and APRI. These biomarkers were found to be correlated with liver fibrosis in patients with chronic hepatitis [11–13]. FIB-4 and APRI have been recommended by the World Health Organization (WHO) in the evaluation of CHB patients [7]. However, the use of FIB-4 and APRI in distinguishing between fibrosis stages has not been well established, and only a few studies have addressed their performance in CHB patients [14, 15].

Nomograms are graphical representations of predictive statistical models for individual patients and have been proposed as an alternative method or even as a new standard for various types of diseases [16–18]. This study aimed to construct a novel nomogram for a more reliable prediction of significant liver fibrosis (*S* ≥ 2) in CHB patients.

## 2. Patients and Methods

### 2.1. Subjects

Between June 2016 and March 2018, 420 consecutive CHB patients with liver biopsy results were included in this study from the Third Affiliated Hospital of Sun Yat-sen University. A total of 252 patients were enrolled between June 2016 and June 2017 as the training set. The validation set comprised 168 individuals between July 2017 and March 2018.

The inclusion criteria were as follows: (1) age range from 18 to 65 years, (2) HBV surface antigen- (HBsAg-) positive for more than 6 months without receiving antiviral treatment before this study, (3) liver biopsy test, and (4) written informed consent.

The exclusion criteria were as follows: (1) co-infection with hepatitis A virus (HAV), hepatitis C virus (HCV), hepatitis D virus (HDV), hepatitis E virus (HEV), or human immunodeficiency virus (HIV); (2) alcoholic or nonalcoholic fatty liver disease; (3) autoimmune liver disease; (4) decompensated cirrhosis; (5) hepatocellular carcinoma; and (6) pregnancy.

This work was carried out in strict accordance with the research design approved by the Clinical Research Ethics Committee of the Third Affiliated Hospital of Sun Yat-sen University, Guangzhou, China.

### 2.2. Clinical Laboratory Parameters

Clinical laboratory parameters were measured and recorded on admission, including age, sex, alanine aminotransferase (ALT), aspartate aminotransferase (AST), PLT, HA, PIIINP, LN and IVC. All of these parameters were measured within 1 week before liver biopsy. Biochemical tests were performed using an automated biochemical analyzer (Hitachi 7600, Tokyo, Japan), and the reagents were provided by Maccura, Sichuan, China. The PLT was determined by an automated blood cell analyzer (Sysmex XN-2000, Kobe, Japan), and the regents were provided by Sysmex, Shanghai, China. The concentrations of the 4 serum markers HA, LN, IVC, and PIIINP were determined by electrochemiluminescence immunoassay (Mindray CL-i2000, Shenzhen, China), and the reagents were provided by Mindray, Shenzhen, China. From these laboratory values, FIB-4 and APRI were calculated exactly as originally described [8, 19].

(1) $$\begin{array}{c} FIB-4^{\left[19\right]}=Age\;\left(years\right)\times AST\;\left(U/L\right)/Platelet\;count\;\left(10^{9}/L\right)\times {\left[ALT\;\left(U/L\right)\right]}^{1/2}\\ APRI^{\left[8\right]}=AST\;level\;\left(/ULN\right)/Platelet\;count\;\left(10^{9}/L\right)\times 100 \end{array}$$

where ULN is the upper limit of normal for that laboratory.

Patients with incomplete data were excluded from this study.

### 2.3. Liver Biopsy

Percutaneous liver biopsies were done under ultrasound guidance. These specimens were fixed in formalin, embedded in paraffin, stained with HE, and histologically assessed by reticulin staining or Masson's trichrome. The inflammation grade (G0–G4) and fibrosis stage (S0–S4) of liver biopsy samples were evaluated based on a modified Scheuer scoring system by two independent experienced pathologists. G0–1 and S0–1 were referred to as no or mild inflammation and fibrosis, respectively; G2–4 and S2–4 were referred to as moderate-to-severe inflammation and fibrosis, respectively. Therefore, patients with fibrosis stage ≥2 (*S* ≥ 2) were classified as having significant liver fibrosis. Inconsistent results were rechecked by pathologists to reach consensus.

### 2.4. Statistical Analysis

Statistical analysis and graphics were performed using SPSS version 15.0 (SPSS, Inc., Chicago, IL). Categorical variables were expressed as frequency, and Fisher's exact test was performed to analyze significance. Continuous variables were expressed as mean ± standard error(SD), or median (interquartile range [IQR]), as appropriate. Parametric test (*t*-test) and nonparametric test (Mann–Whitney *U* test) were used for continuous variables with or without normal distribution, respectively. Univariate and multivariate logistic regression analyses were used to screen the predictors of significant liver fibrosis (*S* ≥ 2) in CHB patients. Independent predictors (*P* < 0.05) in the multivariate logistic regression analysis were included in the nomogram construction. Support vector machine (SVM) was a powerful technique for general classification, regression and outlier detection represented by intuitive model. The rpart programs established classification or regression models of a very general structure, and the resulting models could be indicated as binary trees.

A nomogram is a simple graphical representation of a predictive model that produces numerical probabilities of clinical events. Nomograms for independent predictors associated with significant liver fibrosis (*S* ≥ 2) based on the multivariate logistic regression analyses model from the training set were established with the *rms* package in R version 3.4.0 (http://www.r-project.org/). The use of the nomogram: an individual patient's value is situated on each variable axis, and drawing an upward line gets the number of points for each variable value. Then the sum of these scores is situated on the total points' axis, and drawing a downward line gets the probability of diagnosing significant liver fibrosis (*S* ≥ 2). The nomogram was validated internally in the training set and externally in the validation set. The internal validation was performed by the calibration method and ROC (receiver operating characteristic) curves. The AUROC was calculated. The external validation was performed by calculating the AUROC. The calibration plot with bootstrapping was applied to determine the association between actual probability and predicted probability. Comparisons between the nomogram, FIB-4, and APRI were performed by AUROC. Decision curve analysis is of particular value when the purpose of a model is to help doctors make better clinical decisions. Decision curves were plotted to describe the net benefit given by the nomogram, FIB-4, and APRI. The net benefit is useful to determine whether clinical decisions making on a model would do more benefit than harm. The larger the AUROC was, the more accurate the prediction. A value of *P* < 0.05 was considered statistically significant.

## 3. Results

### 3.1. Clinical Laboratory Parameters of Patients

In the training set, all 252 CHB patients who met the inclusion criteria were enrolled. For the validation set, we studied 168 patients. The clinical laboratory parameters of patients in the training and validation sets are listed in Table 1. Overall, there were no significant differences between the training and validation sets with respect to all variables except age, HA, FIB-4, APRI, liver biopsy (*G* ≥ 2/*G* < 2), and liver biopsy (*S* ≥ 2/*S* < 2).

### 3.2. Univariate and Multivariate Logistic Regression Analysis

The results of the univariate logistic regression analysis are listed in Table 2 and include age, sex, ALT, AST, PLT, HA, PIIINP, LN, IVC, FIB-4, and APRI. Among them, ALT, AST, PLT, HA, PIIINP, LN, IVC, FIB-4, and APRI were statistically significant (*P* < 0.05) in the univariate logistic regression analysis and were included in the multivariate logistic regression analysis. The multivariate logistic regression analysis demonstrated that PLT, LN, and PIIINP were independent predictors of significant liver fibrosis (*S* ≥ 2) in CHB patients (Table 2). In addition, we used three models (logistic, SVM, and rpart) for predicting significant liver fibrosis (*S* ≥ 2) in CHB patients. The results are shown in Supplementary Data (Figures S1, S2, and S3) and confirm that the predictive power of the logistic model was better than the other two models.

### 3.3. Prediction Nomogram for Significant Liver Fibrosis (*S* ≥ 2) in CHB Patients

**Figure 1:**
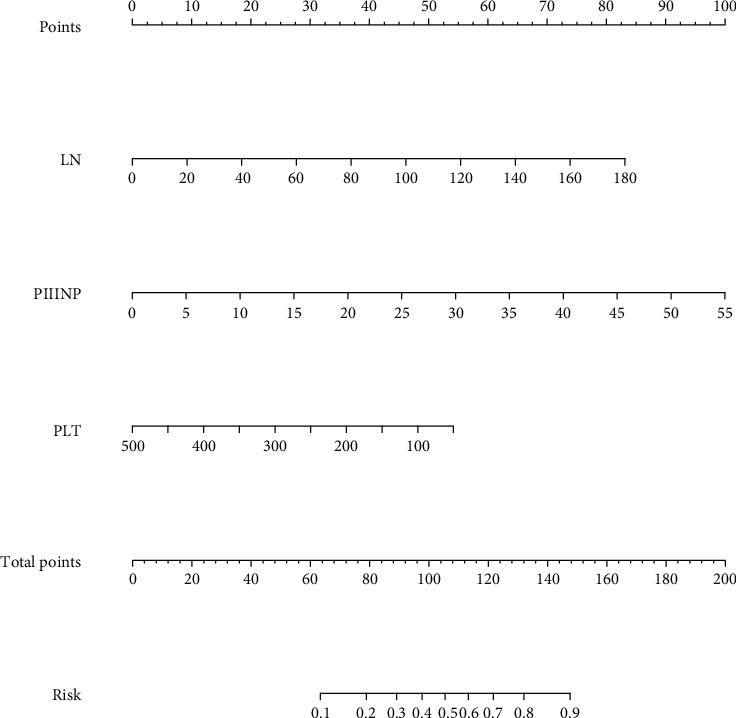
Predictive nomogram. The nomogram for diagnosing significant liver fibrosis (*S* ≥ 2) in CHB patients includes LN, PIIINP and PLT. The use of the nomogram: an individual patient's value is situated on each variable axis, and drawing an upward line gets the number of points for each variable value. Then the sum of these scores is situated on the total points' axis, and drawing a downward line gets the probability of diagnosing significant liver fibrosis (*S* ≥ 2).

**Figure 2:**
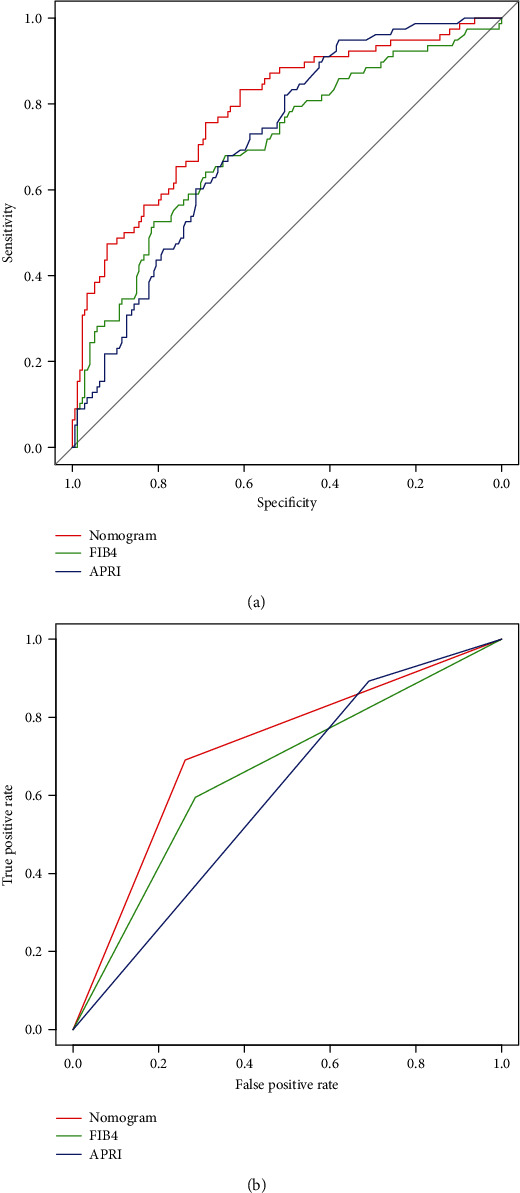
Validation of the nomogram. (a) Internal validation using the ROC curve from the training set. The AUROC is 0.765 (95% CI, 0.694 to 0.835). (b) External validation using the ROC curve from the validation set. The AUROC is 0.714. The AUROCs of the prediction nomogram were higher than that of FIB-4 and APRI in the training set and validation set. ROC: receiver operating characteristic; AUROC: the area under the ROC curve.

**Figure 3:**
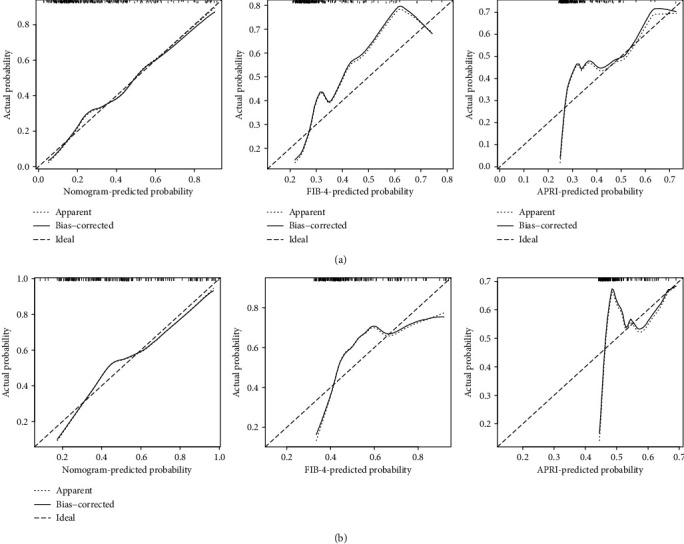
Calibration plot of the prediction nomogram. The nomogram, FIB-4, and APRI were calibrated for the probability of significant liver fibrosis (*S* ≥ 2) in CHB patients (a) in the training set and (b) in the validation set. The predicted probability of significant liver fibrosis (*S* ≥ 2) in CHB patients is plotted on the *x*-axis; the actual probability of significant liver fibrosis (*S* ≥ 2) in CHB patients is plotted on the *y*-axis (bootstrap 1,000 repetitions).

To predict significant liver fibrosis (*S* ≥ 2) in CHB patients, a predictive nomogram was constructed according to all the significant independent indicators determined by the multivariate logistic regression analysis (Figure 1). A total score could be calculated as the sum of scores of the associated predictors and referred to as the probability of significant liver fibrosis in the bottom axis. We used the ROC to compare the accuracy of the nomogram, FIB-4, and APRI for diagnosing significant liver fibrosis (*S* ≥ 2) in CHB patients (Figures 2(a) and 2(b)). The ROC curve showed that the prediction of the nomogram was more consistent with the results of liver biopsy than FIB-4 and APRI. The discrimination abilities of FIB-4 and APRI were unsatisfactory for diagnosing significant liver fibrosis (*S* ≥ 2) (AUROC = 0.673 for FIB-4 and AUROC = 0.640 for APRI) in the training set. However, the AUROC of the nomogram was 0.765 (95% CI, 0.694 to 0.835), which was higher than FIB-4 and APRI and showed the best discrimination power for diagnosing significant liver fibrosis (*S* ≥ 2) in CHB patients. The calibration plot for the probability of significant liver fibrosis (*S* ≥ 2) showed optimal agreement between the prediction by the nomogram and the actual observation (Figure 3(a)).

### 3.4. Validation of the Predictive Accuracy of Nomogram

**Figure 4:**
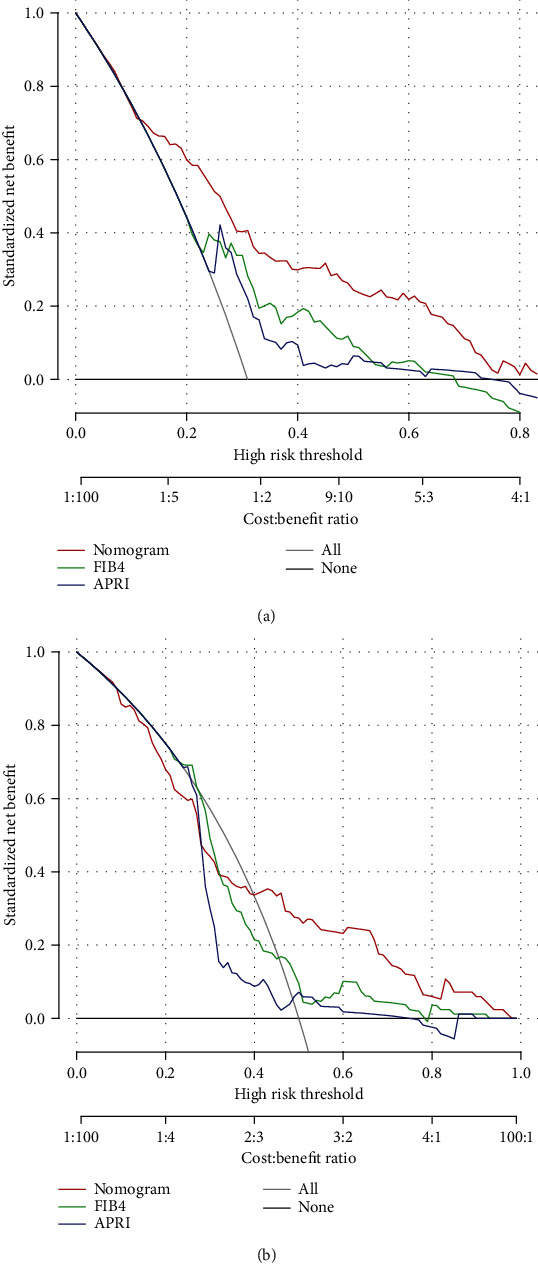
Decision curves for prediction of the net benefit of the constructed nomogram, FIB-4, and APRI. Solid gray line: a perfect prediction model; horizontal solid black line: screen none; red line: screen based on the nomogram; green line: screen based on the FIB-4; blue line: screen based on the APRI. (a) The net benefit of screen patients in the training set according to the nomogram, FIB-4, and APRI. (b) The net benefit of screen patients in the validation set according to the nomogram, FIB-4, and APRI.

In the validation set, the calibration curve showed optimal agreement between the prediction and observation of the probability of significant liver fibrosis (*S* ≥ 2) in the nomogram (Figure 3(b)). The AUROC for the nomogram to predict significant liver fibrosis (*S* ≥ 2) (0.714) was higher than that of FIB-4 (0.655) and APRI (0.601) (Figure 2(b)). As shown in Figures 2(a) and 2(b), the nomogram was superior to FIB-4 and APRI in diagnosing significant liver fibrosis (*S* ≥ 2) for CHB patients in both sets of patients. Similarly, decision curve analysis showed that the nomogram provided a higher clinical net benefit than FIB-4 and APRI at larger threshold probabilities in both patient sets (Figures 4(a) and 4(b)).

## 4. Discussion

A nomogram is a simple graphical representation of a predictive model that produces numerical probabilities of clinical events. In the present study, we successfully established a new nomogram for predicting significant liver fibrosis (*S* ≥ 2) in CHB patients and compared the predictive accuracy of the nomogram with FIB-4 and APRI. Our results demonstrated that our nomogram, comprising PLT, LN, and PIIINP, was more consistent with the observed results from liver biopsies and outperformed FIB-4 and APRI in diagnosing significant liver fibrosis (*S* ≥ 2) in CHB patients.

We combined significant predictive indicators based on the multivariate logistic regression analysis and constructed a nomogram for assessing significant liver fibrosis (*S* ≥ 2) in the training set. We certified the good performance of the nomogram for predicting significant liver fibrosis (*S* ≥ 2) in CHB patients, with an AUROC of 0.765 in the training set and 0.714 in the validation set. The AUROC of the nomogram (0.765) was significantly higher than that of FIB-4 (0.673) and APRI (0.640) in the training set; and similar results were obtained in the validation set.

Prediction models are traditionally evaluated using statistics such as sensitivity and specificity. However, such statistics do not tell us whether the model would do more good than harm if used in clinical practice [20]. Our study attempts to use decision curves to evaluate whether a clinical use of the nomogram would do more benefit than harm, and these decision curves were plotted to describe the net benefit given by the nomogram, FIB-4, and APRI. The results showed that the most net benefits were provided by the nomogram. In addition, this study was the first to evaluate this noninvasive tool in a large number of Chinese CHB patients. We deem that this nomogram will be very practical for predicting significant liver fibrosis (*S* ≥ 2) in clinical practice and could be the foundation of individualized precision therapy.

More than 350 million people worldwide are affected by hepatitis B virus, which is still the main cause of chronic disease and liver-related morbidity in the world [21]. It is imperative to identify those individuals most at risk for liver-related morbidity and mortality and to institute appropriate interventions [22]. Liver fibrosis is considered to be the major risk factor for cirrhosis, liver cancer, and ultimately liver-related death [23, 24]. Since the progress of CHB to fibrosis is reversible, precisely predicting the stage of liver fibrosis is a key feature of a trial designed to assess liver fibrosis.

Although liver biopsy remains the gold standard for diagnosing liver fibrosis, it has many limitations and is not realistic to perform on all CHB patients. Diagnosis of liver fibrosis using various biomarkers, scoring systems, and imaging methods, such as FibroScan, has recently been attempted [25–27]. Nevertheless, the present methods are less than ideal for assessing significant liver fibrosis in CHB patients. Therefore, a more simple, low-cost, useful, and noninvasive model for predicting liver significant fibrosis is urgently needed.

The WHO has recommended FIB-4 and APRI for evaluating CHB patients. These two biomarkers are two noninvasive tools that have been extensively studied for evaluating liver fibrosis in CHB patients [28, 29]. In a meta-analysis of 18 studies, APRI > 0.5 was found to have a sensitivity of 81% and specificity of 55% (AUROC = 0.77) for diagnosing fibrosis [30]. In our study, we evaluated the performance of FIB-4 and APRI on significant liver fibrosis (S ≥2), showing AUROCs of 0.673 and 0.640 in the training set, which was similar to previous studies. The performances of FIB-4 and APRI for diagnosing *F* ≥ 2 were found by Jia et al., who studied 469 CHB patients, with AUROCs of 0.71 and 0.69, respectively [3]. Compared to the 2 scores, the nomogram performed better in diagnosing significant liver fibrosis (*S* ≥ 2) for CHB patients. The AUROC of the nomogram was 0.765 (CI 0.694-0.835). According to the data from the validation set, we discovered that our nomogram showed a modest performance for diagnosing significant liver fibrosis (*S* ≥ 2) with an AUROC of 0.714, compared with FIB-4 (0.655) and APRI (0.601). FIB-4 and APRI have previously been proven to be useful for staging liver fibrosis. However, our results showed that both panels were significantly inferior to our nomogram in CHB patients. Furthermore, the decision curve analysis showed that using a nomogram scoring system in both sets could achieve the maximum net benefit.

Last, although our nomogram represented a useful tool for clinicians to assess significant liver fibrosis (*S* ≥ 2) and select treatment strategies earlier for CHB patients, our study had some limitations. First, the number of cases with liver biopsies was relatively small with the retrospective nature of the study. Second, the samples were obtained from a single institution, and there may be a potential source for selection bias. Thus, further prospective validation studies are warranted to confirm the suitability of this nomogram for clinical practice.

## 5. Conclusions

In conclusion, a novel nomogram with LN, PIIINP, and PLT was constructed in our study, and this noninvasive model showed good consistency with the liver biopsy results and could be recommended as a more accurate and helpful model to predict significant liver fibrosis (*S* ≥ 2) in CHB patients. We recommend this noninvasive assessment for patients with liver fibrosis to avoid the risk of liver biopsy and earlier intervention to prevent the development of cirrhosis or liver cancer for CHB patients.

## Figures and Tables

**Table 1:** Parameters of patients with chronic hepatitis B.

	Training set (*n* = 252)	Validation set (*n* = 168)	*P*
Age (year)	38.59 ± 12.36	42.76 ± 16.66	0.003
Gender (male/female)	160/92	98/70	0.288
ALT (U/L)	46.0 (26.0-114.5)	49.0 (24.0-101.0)	0.841
AST (U/L)	36.0 (24.0-64.75)	38.0 (25.0-71.75)	0.406
TBIL (*μ*mol/L)	14.5 (10.6-18.8)	14.0 (8.9-23.1)	0.803
ALB (g/L)	45.4 (41.4-47.2)	43.4 (41.5-46.1)	0.363
PLT (×10^9^/L)	186.0 (151.0-232.0)	183.0 (133.25-226.5)	0.188
HA (ng/mL)	40.98 (26.08-72.19)	48.16 (31.56-91.28)	0.016
LN (ng/mL)	53.03 (41.91-65.00)	49.73 (41.62-62.35)	0.266
IVC (ng/mL)	48.72 (28.79-81.04)	56.99 (33.63-106.02)	0.115
PIIINP (ng/mL)	10.77 (7.39-17.43)	11.04 (7.85-16.8)	0.472
HBeAg (+/-)	91/161	42/126	
HBeAb (+/-)	119/133	60/108	
Inflamation,G0/G1/G2/G3/G4, *n*	28/130/70/23/1	11/75/66/15/1	
Liver biopsy (*G* ≥ 2/*G* < 2)	94/158	82/86	0.021
Stage of fibrosis, S0/S1/S2/S3/S4, *n*	35/138/46/23/10	13/72/44/33/6	
Liver biopsy (*S* ≥ 2/*S* < 2)	79/173	83/85	0.001

ALT: alanine aminotransferase; AST: aspartate aminotransferase; TBIL: total bilirubin; ALB: albumin; PLT: blood platelet; HA: hyalurona; LN: laminin; IVC: type IV collagen; PIIINP: procollagen type III N-terminal peptide; HBeAg: hepatitis B e antigen; HBeAb: hepatitis B e antibody; *S* ≥ 2: significant liver fibrosis; *S* < 2: no or mild liver fibrosis.

**Table 2:** Univariate and Multivariate Analysis of Predictors of the training set.

Variable	Univariate		Multivariate	
	OR (95% CI)	*P*	OR (95% CI)	*P*
Age (year)	1.020 (0.998-1.042)	0.077		
Gender (male/female)	1.159 (0.664-2.024)	0.604		
ALT (U/L)	1.002 (1.000-1.004)	0.025		
AST (U/L)	1.005 (1.001-1.008)	0.007		
PLT (×10^9^/L)	0.993 (0.989-0.998)	0.005	0.993 (0.989-0.998)	0.008
HA (ng/mL)	1.015 (1.008-1.021)	<0.001		
LN (ng/mL)	1.0332 (1.017-1.047)	<0.001	1.024 (1.008-1.041)	0.003
IVC (ng/mL)	1.011 (1.007-1.016)	<0.001		
PIIINP (ng/mL)	1.112 (1.071-1.154)	<0.001	1.095 (1.055-1.138)	<0.001
FIB4	1.533 (1.214-1.936)	<0.001		
APRI	1.700 (1.246-2.322)	0.001		

ALT: alanine aminotransferase; AST: aspartate aminotransferase; PLT: blood platelet; HA: hyalurona; LN: laminin; IVC: type IV collagen; PIIINP: procollagen type III N-terminal peptide; FIB-4: fibrosis 4 score; APRI, AST: platelet ratio index; OR: odds ratio; 95% CI: 95% confidence interval. Multivariate analysis was adjusted for age, ALT, AST, PLT, HA, LN, IVC, and PIIINP.

## Data Availability

The data that support the findings of this study are available upon reasonable request from the corresponding author. Data requests should be made to Bo Hu, hubo@mail.sysu.edu.cn.

## Supplementary Materials

Supplementary Materials Figure S1: the ROC curve of the three models logistic, SVM, and rpart for predicting significant liver fibrosis (*S* ≥ 2) in patients with chronic hepatitis B. Figure S2: the predictive power of the three models logistic, SVM, and rpart for significant liver fibrosis (*S* ≥ 2) in patients with chronic hepatitis B. Figure S3: 95% confidence interval under the ROC curve of the three models logistic, SVM, and rpart for predicting significant liver fibrosis (*S* ≥ 2) in patients with chronic hepatitis B.
