# Supplementary material for: A Novel Prediction Model for Significant Liver Fibrosis in Patients with Chronic Hepatitis B
Source: Biomed Res Int. 2020 Jul 8;2020:6839137. doi: 10.1155/2020/6839137 (PMC7368191; doi:10.1155/2020/6839137)
Supplement: Supplementary Materials — Figure S1: the ROC curve of the three models logistic, SVM, and rpart for predicting significant liver fibrosis (S ≥ 2) in patients with chronic hepatitis B. Figure S2: the predictive power of the three models logistic, SVM, and rpart for significant liver fibrosis (S ≥ 2) in patients with chronic hepatitis B. Figure S3: 95% confidence interval under the ROC curve of the three models logistic, SVM, and rpart for predicting significant liver fibrosis (S ≥ 2) in patients with chronic hepatitis B. [file 6839137.f1.pdf]

Supplementary Material:

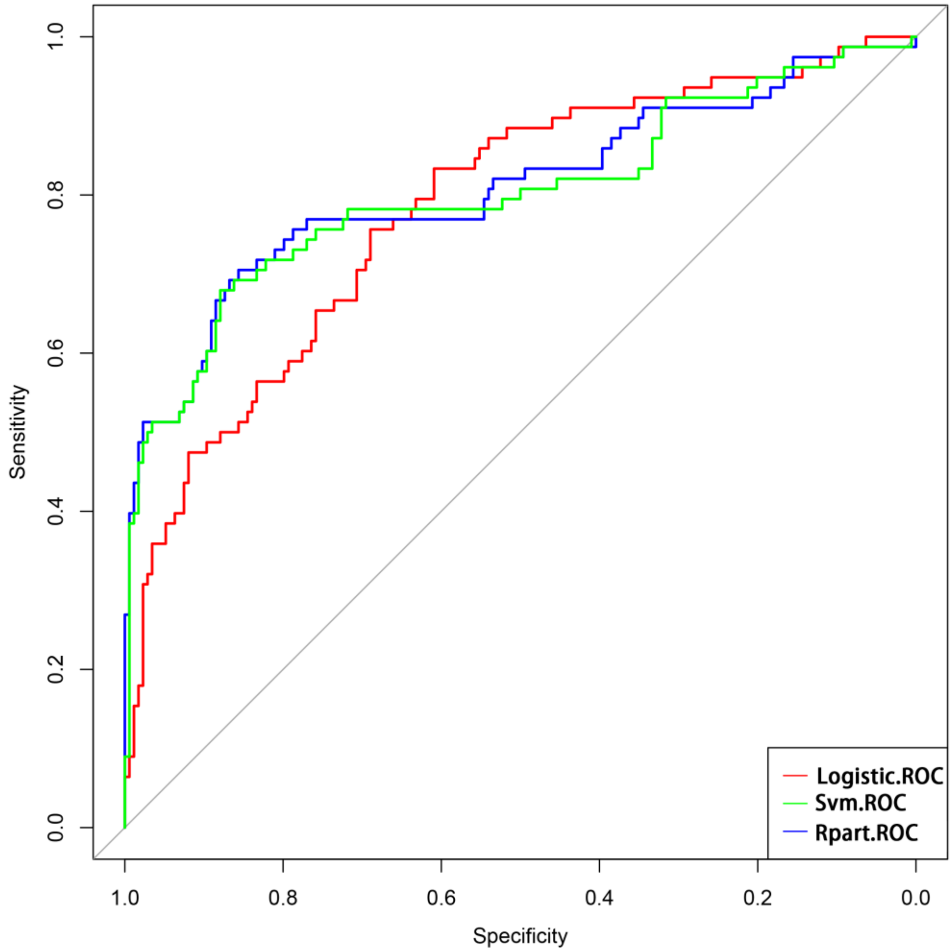

Figure S1. The ROC curve of logistic, svm, and rpart three models for predicting significantliver fibrosis(S≥2) in patients with chronic hepatitis B. The AUROC of the logistic model was significantly higher than that of svm, and rpart models.

Svm, support vector machine; ROC, receiver operating characteristic; AUROC, the area under the ROC curve.

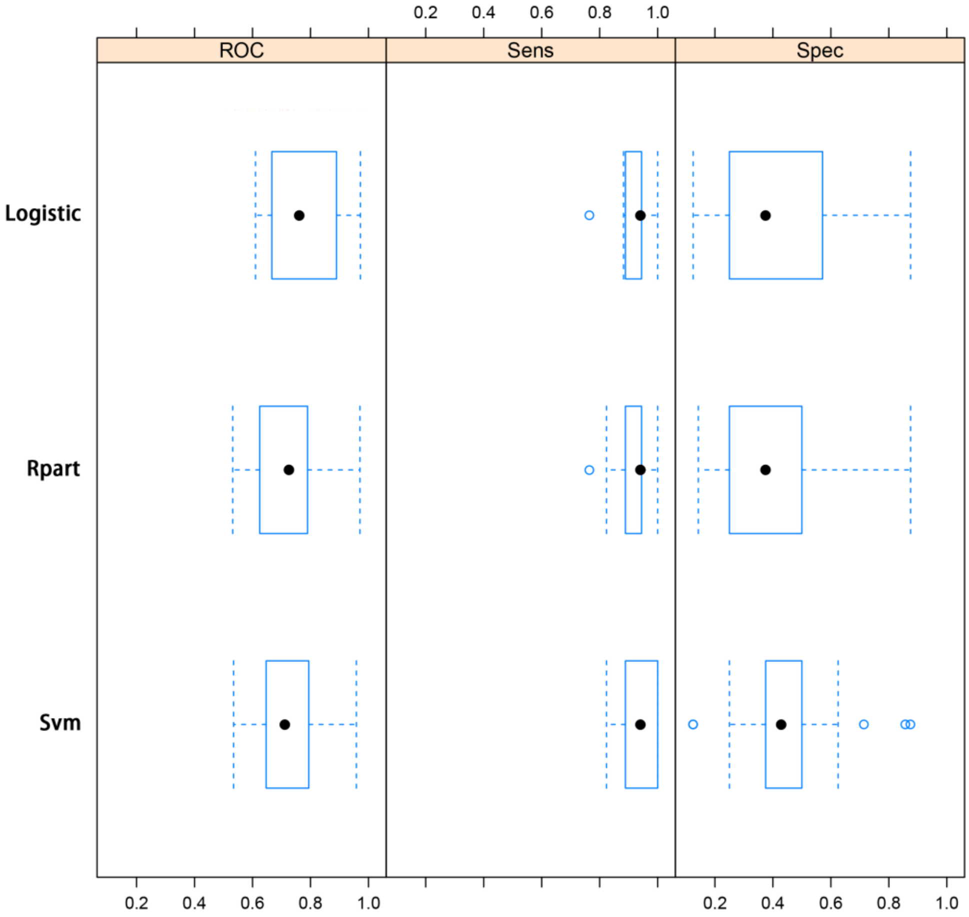

Figure S2. The predictive power of logistic, svm, and rpart three models for significant liver fibrosis(S≥2) in patients with chronic hepatitis B. The AUROC, sensitivity and specificity of logistic were all significantly higher than that of svm, and rpart models.

AUROC, the area under the ROC curve.

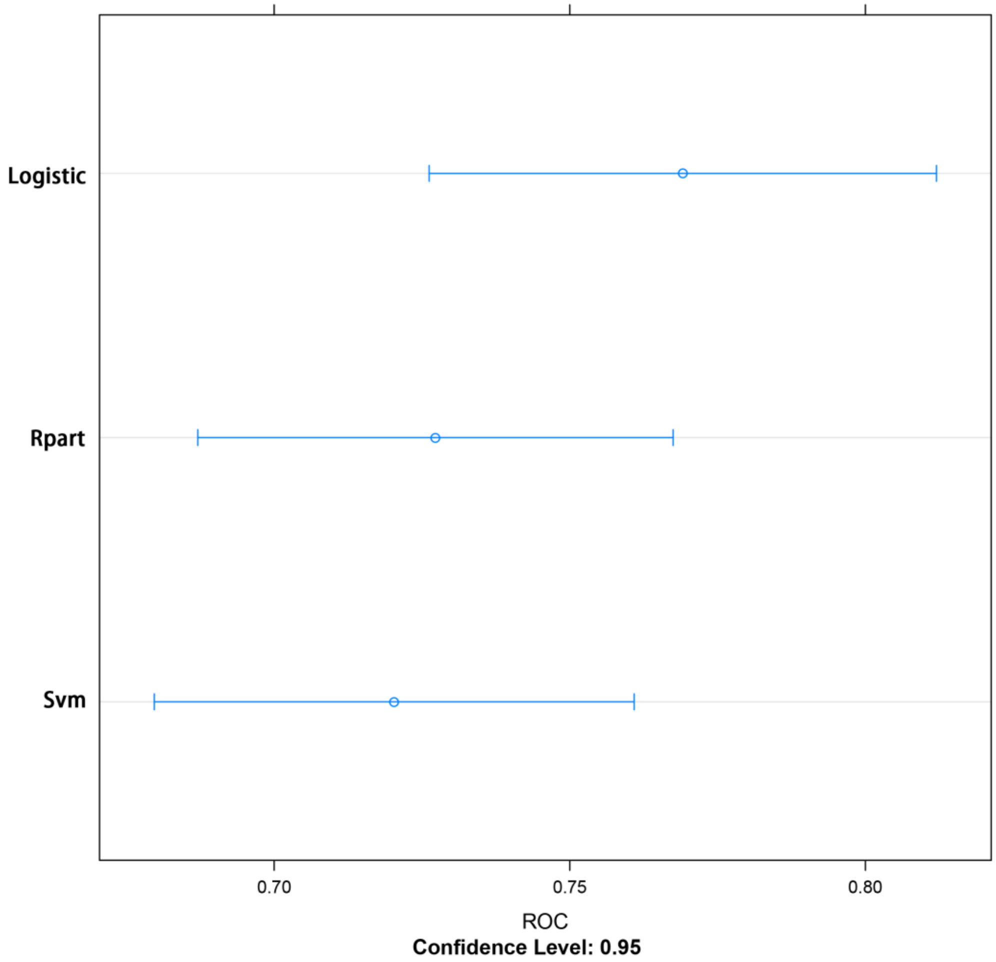

Figure S3. 95% confidence interval under the ROC curve of logistic, svm, and rpart.

Three models for predicting significant liver fibrosis(S≥2) in patients with chronic hepatitis B.
